# Surgical repair of pseudoaneurysms and complex arteriovenous fistula between popliteal vessels

**DOI:** 10.1590/1677-5449.000618

**Published:** 2018

**Authors:** Adenauer Marinho de Oliveira Góes, Carolina Pinheiro de Oliveira, Camilla Castilho Maia, Bruno Campos Xavier, Silvia Karinny Brito Calandrini de Azevedo

**Affiliations:** 1 Centro Universitário do Estado do Pará (CESUPA), Belém, PA, Brasil.; 2 Hospital Metropolitano de Urgência e Emergência (HMUE), Ananindeua, PA, Brasil.

**Keywords:** arteriovenous fistula, wounds and injuries, popliteal artery, popliteal vein, aneurysm, false, surgery

## Abstract

An arteriovenous fistula (AVF) is an abnormal and permanent communication between an artery and a vein caused by penetrating traumas or iatrogenic injuries. A penetrating trauma to the endothelial wall can lead to formation of pseudoaneurysms (PSA) and to formation of an AVF. Here, the authors present the case of a patient with a complex AVF of popliteal vessels, associated with popliteal artery pseudoaneurysm, suggested by clinical features and imaging exams, and treated with conventional surgery due to unavailability of a stent graft with appropriate diameter and because endovascular surgery isn’t provided at the service where this patient was operated.

## INTRODUCTION

 An arteriovenous fistula (AVF) is an abnormal and permanent communication between an artery and a vein, 1 ^,^ 2 which is generally associated with penetrating traumas and iatrogenic injuries. 1 ^,^ 3 The duration of clinical presentation and the time that elapses between trauma and diagnosis vary and may even run to decades. 4 Penetrating traumas to the artery wall can cause formation of pseudoaneurysms and, if there is also venous damage, to development of AVF. 1 However, simultaneous occurrence of both clinical conditions is a rare complication that has been described little. 5

 The superficial femorals are the vessels most often involved (22%), followed by the popliteal vessels (16%). 6 Trauma to popliteal vessels involves significant risk of amputation. 7 An AVF is primarily diagnosed on the basis of clinical status, with localized murmurs and thrills, edema, and venous ulcers. Diagnostic investigation tends to be pursued using imaging exams, such as Doppler echography, angiotomography, and sometimes angiography. 1

### Part I – clinical situation

 The patient was a 53-year-old male who had been wounded in the left thigh by a cartridge belt 2 years and 9 months previously. 

 The patient complained of pain in the left lower limb, which had developed edema, varicose veins, ochrodermatitis and ulceration of the anterior surface of the leg. The limb involved had no palpable distal pulses and thrill and murmur were detectable from the groin to the proximal third of the leg (with greatest intensity in the popliteal fossa). Pulsation was also noted along the entire length of the patient’s thigh. 

**Figure 1:**
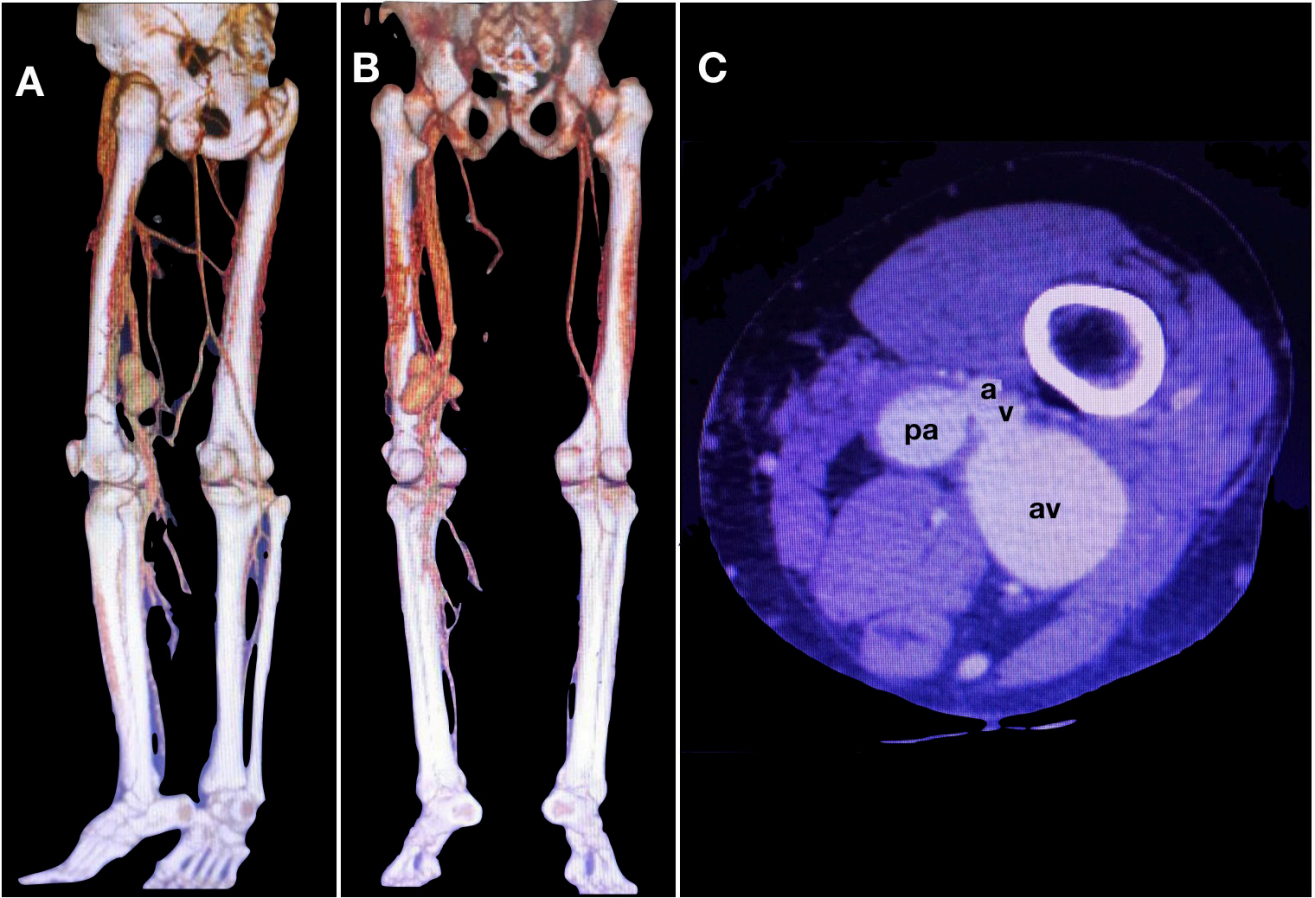
(A) and (B) Angiotomography reconstructions from different angles, showing simultaneous impregnation of the arterial system and the deep vein system in the left thigh by contrast and large dilatations of the popliteal vessels; (C) Axial computed tomography of the left lower limb; a: popliteal artery; pa: pseudoaneursym of the popliteal artery; v: popliteal vein; av: aneurysm of the popliteal vein.

 Angiotomography revealed AVF of the left popliteal vessels, a left popliteal artery pseudoaneurysm with a 2.6 cm diameter and an aneurysm of the popliteal vein with a 5 cm diameter ( Figure 1 ). 

### Part II – what was done

 The decision was taken to perform open surgery to repair the vessels involved. The procedure was conducted under general anesthesia. 

 With the patient in ventral decubitus, with a pneumatic cuff already in place at the base of the left thigh (in case of need for urgent hemostasis), an italic-S incision was made in the left popliteal region, revealing large dilations of the popliteal vessels. Dissection of these vessels was complicated by fibrosis and diffuse bleeding caused by venous hypertension of the limb, but it was not necessary to inflate the pneumatic cuff. The infragenicular segment of the popliteal artery was dissected and repaired, but because of the large volume of the dilatations of the vessels involved, the surgical field was not large enough to obtain proximal control safely. 

 The patient was repositioned in dorsal decubitus, to enable a longitudinal incision to be opened along the medial surfaces of the left thigh and leg and proximal and distal control of the vessels involved was achieved. When the artery was clamped, stopping flow through the fistula, arterial hypotension set in and vasoactive drugs were needed. 

**Figure 2:**
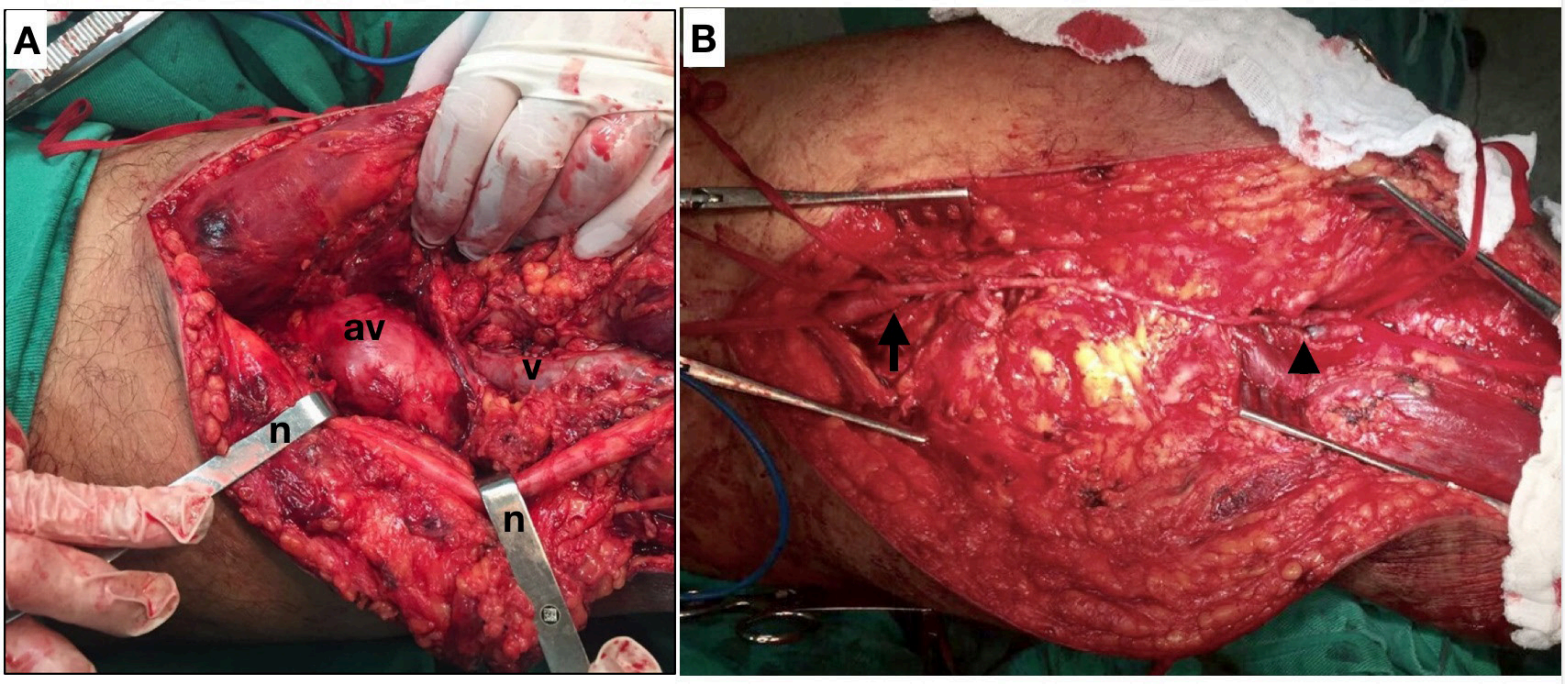
(A) Posterior italic-S surgical access; n: retractors applied to the sciatic nerve; av: aneurysm of the popliteal vein; v: popliteal vein; (B) Medial surgical access; the arrow indicates the proximal anastomosis of the graft to the supragenicular popliteal artery; the arrowhead indicates the distal anastomosis of the graft to the infragenicular popliteal artery.

 Arterial and venous ligatures were performed proximal and distal of the AVF and then a graft was constructed from the supragenicular to infragenicular segments of the popliteal artery using a length of the contralateral great saphenous vein reversed (with proximal end-to-side and distal end-to-end anastomoses). Since the patient was hemodynamically unstable, the operation was concluded without reconstruction of the deep vein system ( Figure 2 ). 

**Figure 3:**
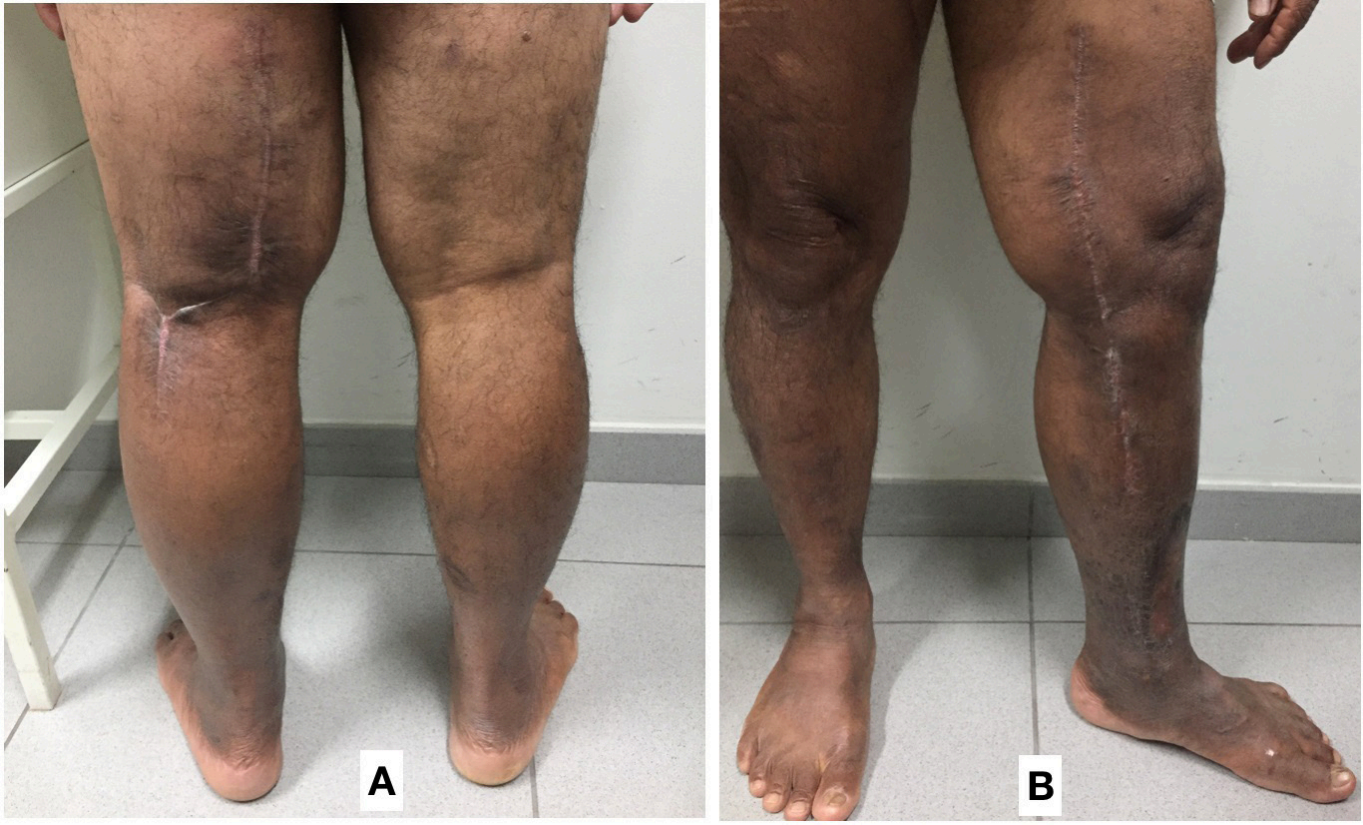
Photographs taken 6 months after the operation, showing the healed surgical scars. (A) Posterior italic-S surgical access; (B) Medial surgical access.

 The patient has been in outpatients follow-up for 6 months. The surgical wounds and the venous ulcer have healed and there has been no further increase in edema of the limb compared to the preoperative baseline and the limb has exhibited satisfactory functional recovery ( Figure 3 ). 

## DISCUSSION

 In high output FAVs diagnosed late, the low resistance flow causes arteriomegaly proximal of the AVF. 8 There are two theories to explain this dilatation. According to the first theory, shear forces resulting from the increased flow rate at the site cause increased production of endothelium-derived relaxing factor, which in turn provokes dilatation of arterial smooth muscle. The second theory states that late increase of blood flow in the artery proximal to the AVF causes destruction of the elastic fibers in this segment, with progression to arteriomegaly. 4

 Many different procedures for repair of pseudoaneurysms and AVFs have been described: aneurysmorrhaphy, resection of the aneurysmal segment and interposition of prosthetic or venous grafts, placement of stent-grafts, and combination procedures. 9

 Endovascular surgery offers the advantages of reduced morbidity and mortality, shorter length of hospital stay, and preservation of the great saphenous vein, 7 although there is still a lack of long-term follow-up studies. 10 However, for treatment of true aneurysms of the popliteal artery, it is known that conventional surgery offers superior long-term patency, particularly in younger patients and when arterial reconstruction is performed using a venous graft. 3

 In the case described here, conventional surgery was chosen. One of the factors that influenced the decision to employ open surgery was that no covered stent with an appropriate diameter was available, 9 because the arteriomegaly proximal to the AVF resulted in a difference in caliber between the artery proximal and distal of the AVF and there is no conical covered stent that could fit the disproportion between these diameters. Possibly, the lack of a suitable conical stent could have been dealt with by releasing multiple covered stents with gradually increasing calibers overlapping each other, using tapered stent-grafts. 11 ^-^ 16 However, the long-term efficacy of this endovascular option is lacking evidence from studies, especially in the topography of the knee joint. Furthermore, endovascular surgery is not available at the service where this patient was treated. 

 In this case, surgical treatment was initiated with a posterior access to the popliteal vessels. Posterior access should be used when the objective is to expose only the segments of the popliteal vessels posterior to the joint. 7 However, the large volume of the venous aneurysm interfered with the arterial dissection procedure and the posterior approach did not provide sufficient access to safely control the proximal artery. The patient was therefore turned over to dorsal decubitus and a medial access was performed, providing good exposure and enabling the initial incision to be extended. 7

 When we analyzed the angiotomography images retrospectively, we concluded that the posterior access should not have been attempted. We recommend that in future cases the level of the lesion in relation to the patella should be used as a reference for planning surgery and that medial access is preferable when the lesion is higher than the upper margin of the patella. 

 In cases such as the one reported here, the priority is arterial reconstruction, which was achieved with a graft constructed using the contralateral great saphenous vein, preserving the saphenous vein in the operated limb for venous return, because of the possible need to ligate the deep vein system. Whenever possible, the deep vein system should also be reconstructed by venorrhaphy, resection, and anastomosis or grafting. Venous ligature should be avoided because of the possibility of chronic venous hypertension of the limb and its medium and long-term clinical repercussions. 1 In the case reported here, popliteal vein ligature was performed because of the risk of thrombosis of the large venous aneurysm and of emboli after the AVF was closed. Additionally, reconstruction with a venous graft, which had been planned initially, was not performed because of hemodynamic instability. 
