# Supplementary material for: Surgical repair of pseudoaneurysms and complex arteriovenous fistula between popliteal vessels
Source: J Vasc Bras. 2018 Jul-Sep;17(3):229–33. doi: 10.1590/1677-5449.000618 (PMC6326132; doi:10.1590/1677-5449.000618)
Supplement: Translation

# Translation: Correção cirúrgica de pseudoaneurismas e fístula arteriovenosa complexa entre vasos poplíteos

**Authors:** Adenauer Marinho de Oliveira Góes, Carolina Pinheiro de Oliveira, Camilla Castilho Maia, Bruno Campos Xavier, Silvia Karinny Brito Calandrini de Azevedo

**Affiliations:** 1 Centro Universitário do Estado do Pará (CESUPA), Belém, PA, Brasil.; 2 Hospital Metropolitano de Urgência e Emergência (HMUE), Ananindeua, PA, Brasil.

**Keywords:** fístula arteriovenosa, ferimentos e lesões, artéria poplítea, veia poplítea, falso aneurisma, cirurgia

## Abstract

A fístula arteriovenosa (FAV) é uma comunicação anormal e permanente entre uma artéria e uma veia devido a traumas penetrantes e lesões iatrogênicas. O trauma penetrante na parede arterial pode levar à formação de pseudoaneurismas (PSA) e, se houver lesão venosa concomitante, à formação de uma FAV. Os autores apresentam o caso de um paciente portador de FAV complexa de vasos poplíteos associada a pseudoaneurisma de artéria poplítea, sugeridos a partir de exames clínicos e exames de imagem, e tratados por cirurgia convencional devido à indisponibilidade de um stent *graft* com diâmetro apropriado, além de a cirurgia endovascular não estar disponível no serviço em que o paciente foi operado.

## INTRODUÇÃO

 A fístula arteriovenosa (FAV) é uma comunicação anormal permanente entre uma artéria e uma veia 1 ^,^ 2 que está comumente associada a traumas penetrantes e lesões iatrogênicas 1 ^,^ 3 . O tempo de apresentação clínica e o período entre o trauma e o diagnóstico é variável, podendo alcançar décadas 4 . O trauma penetrante na parede arterial pode levar à formação de pseudoaneurismas e, se houver lesão venosa concomitante, ao desenvolvimento de FAV 1 . A ocorrência simultânea de ambas as condições clínicas é uma complicação rara e pouco descrita 5 . 

 Os vasos mais acometidos são os femorais superficiais (22%), seguidos dos vasos poplíteos (16%) 6 . O trauma em vasos poplíteos acarreta risco significativo de amputação 7 . A FAV é diagnosticada sobretudo pelo quadro clínico, com sopro e frêmito locais, edema e úlcera venosa. A investigação diagnóstica costuma avançar com exames de imagem, como ecografia Doppler, angiotomografia e eventualmente angiografia 1 . 

### Parte I – situação clínica

 Paciente do sexo masculino, 53 anos, sofreu ferimento por “cartucheira” na coxa esquerda há 2 anos e 9 meses. 

 O paciente evoluiu com dor, edema, varizes, dermatite ocre em membro inferior esquerdo, além de úlcera em face anterior da perna. No membro acometido não havia pulsos distais palpáveis e havia frêmito e sopro perceptíveis desde a região inguinal até o terço proximal da perna (mais intensos na fossa poplítea). Na inspeção chamava atenção a “pulsatilidade” de toda a coxa do paciente. 

 A angiotomografia demonstrou FAV de vasos poplíteos esquerdos, pseudoaneurisma de artéria poplítea esquerda de 2,6 cm de diâmetro e um aneurisma de veia poplítea com 5 cm de diâmetro ( Figura 1 ). 

**Figura 1:** (A) e (B) Reconstruções da angiotomografia em diferentes incidências, evidenciando a impregnação pelo meio de contraste simultaneamente do sistema arterial e do sistema venoso profundo na coxa esquerda e volumosas dilatações dos vasos poplíteos; C) Corte axial da tomografia computadorizada do membro inferior esquerdo; a: artéria poplítea; pa: pseudoaneursima da artéria poplítea; v: veia poplítea; av: aneurisma da veia poplítea.

### Parte II – o que foi feito

 Foi indicada a correção cirúrgica por via aberta. O procedimento foi realizado sob anestesia geral. 

 Com o paciente em decúbito ventral e um manguito pneumático na raiz da coxa esquerda (em caso da necessidade urgente de hemostasia), procedeu-se incisão em “S” itálico na região poplítea esquerda, identificando-se as volumosas dilatações dos vasos poplíteos. A dissecção desses vasos foi prejudicada pela presença de fibrose e sangramento difuso devido à hipertensão venosa do membro, porém não chegou a ser necessária a insuflação do manguito pneumático. A artéria poplítea foi dissecada e reparada no segmento infragenicular; porém, em virtude do grande volume das dilatações dos vasos envolvidos, o campo cirúrgico foi insuficiente para um controle proximal seguro. 

 O paciente foi reposicionado em decúbito dorsal a fim de realizar uma incisão longitudinal nas faces mediais da coxa e perna esquerdas, obtendo-se o controle proximal e distal dos vasos envolvidos. Quando a artéria foi pinçada, interrompendo-se o fluxo para a fístula, houve hipotensão arterial, sendo necessário o uso de drogas vasoativas. 

 Foram realizadas ligaduras arteriais e venosas proximais e distais à FAV e, a seguir, enxerto entre as porções supra e infrageniculares da artéria poplítea com segmento da veia safena magna contralateral reversa (anastomose proximal término-lateral e distal término-terminal). Devido à instabilidade hemodinâmica, a cirurgia foi abreviada sem que o sistema venoso profundo fosse reconstruído ( Figura 2 ). 

**Figura 2:** (A) Acesso cirúrgico posterior em “S” itálico; n: afastadores retraindo o nervo ciático; av: aneurisma da veia poplítea; v: veia poplítea; (B) Acesso cirúrgico medial; a seta indica a anastomose proximal do enxerto na porção supragenicular da artéria poplítea; a ponta de seta indica a anastomose distal do enxerto na porção infragenicular da artéria poplítea.

 O paciente vem sendo acompanhado ambulatorialmente há 6 meses. As feridas operatórias e a úlcera venosa cicatrizaram, não houve aumento do edema do membro em relação ao pré-operatório, e o membro apresentou recuperação funcional satisfatória ( Figura 3 ). 

**Figura 3:** Fotografias feitas no 6º mês pós-operatório mostrando as feridas operatórias cicatrizadas. (A) Acesso cirúrgico posterior em “S” itálico; (B) Acesso cirúrgico medial.

## DISCUSSÃO

 Nas FAVs de alto débito com diagnóstico tardio, o fluxo de baixa resistência promove uma arteriomegalia proximal à FAV 8 . Duas teorias explicam essa dilatação. Na primeira, a força de cisalhamento provocada pelo aumento do fluxo local leva a um aumento na produção do fator de relaxamento derivado do endotélio, provocando uma dilatação da musculatura lisa arterial. A segunda teoria afirma que o aumento tardio do fluxo sanguíneo na artéria proximal à FAV leva à destruição das fibras elásticas desse segmento, ocorrendo a progressão da arteriomegalia 4 . 

 Para a correção de pseudoaneurismas e FAVs, diferentes procedimentos já foram descritos: aneurismorrafia, ressecção de segmento aneurismático e interposição de próteses ou enxertos venosos, implante de *stent-graft* e procedimentos combinados 9 . 

 A cirurgia endovascular tem a vantagem de reduzir a morbimortalidade, o tempo de hospitalização e preservar a veia safena magna 7 , porém seu uso ainda carece de estudos com acompanhamento a longo prazo 10 . Entretanto, para o tratamento de aneurismas verdadeiros da artéria poplítea, sabe-se que a cirurgia convencional proporciona perviedade superior a longo prazo, principalmente em pacientes jovens e quando a reconstrução arterial é feita com enxerto de veia 3 . 

 No caso aqui relatado, optou-se pela realização da cirurgia convencional. Um dos fatores que influenciou a opção pela cirurgia aberta foi a indisponibilidade de um stent revestido com diâmetro apropriado 9 , uma vez que a arteriomegalia proximal à FAV gerou uma diferença de calibres entre a artéria proximal e distal à FAV, e não há um stent revestido cônico adequado à desproporção dos diâmetros. A indisponibilidade de um stent cônico poderia, eventualmente, ser contornada pela liberação de múltiplos stents revestidos de calibres gradualmente crescentes em *overlapping* utilizando *tapered stent grafts* 11 ^-^ 16 . Porém, essa alternativa endovascular, especialmente na topografia da articulação do joelho, carece de estudos que comprovem sua eficácia a longo prazo; além disso, a cirurgia endovascular não está disponível no serviço em que o paciente foi operado. 

 No presente caso, o tratamento cirúrgico foi iniciado com acesso posterior para os vasos poplíteos. O acesso posterior deve ser realizado quando o objetivo é a exposição apenas dos segmentos retroarticulares dos vasos poplíteos 7 . Porém, o grande volume do aneurisma venoso prejudicou a dissecção arterial, e esse acesso não possibilitou controle arterial proximal com segurança. Por isso, o decúbito do paciente foi alterado e o acesso medial foi realizado, o que permitiu uma boa exposição e a possibilidade de estender a incisão inicial 7 . 

 Ao analisar as imagens da angiotomografia retrospectivamente, concluímos que o acesso posterior não deveria ter sido realizado. Recomendamos que a altura da lesão em relação à patela seja usada como referência para o planejamento de casos futuros; quando a lesão estiver localizada acima da borda superior da patela, o acesso medial é o mais indicado. 

 Em casos como o aqui relatado, a prioridade é a reconstrução arterial, o que foi feito com enxerto usando a veia safena magna contralateral, preservando-se a safena do membro operado para o retorno venoso, pois a ligadura do sistema venoso profundo pode ser necessária. Sempre que possível, a reconstrução do sistema venoso profundo também deve ser realizada por venorrafia, ressecção e anastomose ou enxerto. A ligadura venosa deve ser evitada, devido à possibilidade de o membro evoluir com hipertensão venosa crônica e suas repercussões clínicas no curto e médio prazo 1 . No caso aqui relatado, a ligadura da veia poplítea foi realizada devido ao risco de trombose do volumoso aneurisma venoso e embolia após a desativação da FAV; além disso, devido à instabilidade hemodinâmica, a reconstrução com enxerto venoso inicialmente planejada não foi realizada.
